# Curious Case of Hypertrophy of the Muscular Structure of the Stomach

**Published:** 1830-10-01

**Authors:** 


HYPERTROPHY OF THE MUSCULAR COAT OF THE STOMACH, &c. By M. REYNAUD.

CASE. A labourer, aged 58 years, of robust constitution, was seized, towards the end of May, 1828, with symptoms of the endemic which then prevailed in Paris. For two or three months this man had complained of loss of appetite, urgent thirst, pains in his limbs and arms, which last had diminished, but never entirely disappeared, and were succeeded by paralysis of the lower extremities. In fact, for three months before his death he was totally incapable of walking. When he came under the reporter's care, the hands and arms were still the seat of darting pains, and they were contracted and distorted. He now also complained of constant pain at the pit of the stomach and under the sternum. His appetite had been quite gone for a month. The epigastrium offered great resistance on pressure—the vomitings were frequent—the tongue was moist and natural—thirst considerable — obstinate constipation—skin dry—pulse slightly accelerated — emaciation advanced. This emaciation and these symptoms continued till the 3d of April, when the patient died.

*Dissection*. Passing over the minute but useless details of dissection respecting the head and thorax, we find that the stomach was more ample than natural, and had contracted some adhesions with the liver. The organ contained some undigested matters, and about two pints of a dark-coloured fluid. The mucous membrane was rather thinner than natural. The pyloric orifice would scarcely admit the point of the little finger. There was a thickened and *scirrhous* state of the cellular substance interposed between the coats of the stomach around the pylorus for a little way. But the whole parietes of the organ were in a state of great hypertrophy. This hypertrophy was seated in the muscular structure of the stomach, which was as red as the fibres of any of the voluntary muscles.

The lesions which were observed in the cortical substance of the brain, were supposed to account for the loss of sense and motion in the lower extremities; but as they did not appear satisfactory to us, we shall not occupy space with them here. The hypertrophy of the muscular coat of the stomach is curious and interesting. HEBDOM.

---

XXVI.

